# NME6: ribonucleotide salvage sustains mitochondrial transcription

**DOI:** 10.15252/embj.2023114990

**Published:** 2023-08-07

**Authors:** Paulina H Wanrooij, Andrei Chabes

**Affiliations:** ^1^ Department of Medical Biochemistry and Biophysics Umeå University Umeå Sweden

**Keywords:** Organelles, RNA Biology

## Abstract

The building blocks for RNA and DNA are made in the cytosol, meaning mitochondria depend on the import and salvage of ribonucleoside triphosphates (rNTPs) and deoxyribonucleoside triphosphates (dNTPs) for the synthesis of their own genetic material. While extensive research has focused on mitochondrial dNTP homeostasis due to its defects being associated with various mitochondrial DNA (mtDNA) depletion and deletion syndromes, the investigation of mitochondrial rNTP homeostasis has received relatively little attention. In this issue of the EMBO Journal, Grotehans *et al* provide compelling evidence of a major role for NME6, a mitochondrial nucleoside diphosphate kinase, in the conversion of pyrimidine ribonucleoside diphosphates into the corresponding triphosphates. These data also suggest a significant physiological role for NME6, as its absence results in the depletion of mitochondrial transcripts and destabilization of the electron transport chain (Grotehans *et al*, 2023).

## Graphical abstract

A recent study shows that a nucleoside kinase provides the building blocks for mitochondrial transcription.

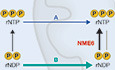

The import of RNA and DNA building blocks can potentially occur at different phosphorylation levels (Fig 1), including (deoxy)ribonucleoside mono‐, di‐, and triphosphates (rNMPs/dNMPs, rNDPs/dNDPs, and rNTPs/dNTPs) or at the level of (deoxy)ribonucleosides (rNs/dNs), which lack the phosphate groups. Before they can be used in mitochondria, rNs/dNs, rNMPs/dNMPs, and rNDPs/dNDPs must be phosphorylated, resulting in their conversion to rNTPs/dNTPs. This process is referred to as salvage.

**Figure 1:**
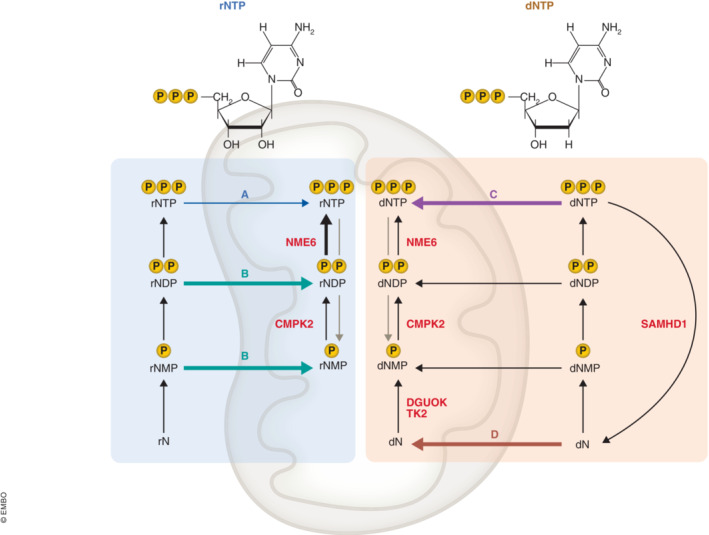
Schematic presentation of (deoxy)ribonucleotide import and salvage in mitochondria The discussed enzymes are indicated; see the text for details.

But at what phosphorylation level do ribonucleotides normally enter mitochondria? Pyrimidine ribonucleotides can enter mitochondria directly as NTPs through two mitochondrial pyrimidine (deoxy)ribonucleotide carriers (SLC25A33 and SLC25A36; Fig 1—arrow A). However, this route is likely to be a minor one under normal conditions, as Grotehans *et al* demonstrated that it was unable to support normal mitochondrial NTP pools in NME6‐deficient cells. Instead, the amount of mitochondrial CTP decreased as CMP increased. This was accompanied by a simultaneous depletion of mitochondrial transcripts in NME6‐depleted cells, indicating that the main pathway for importing pyrimidine ribonucleotides into mitochondria is at the level of NDPs or NMPs, presumably via the same carriers (Fig 1—arrow B). This hypothesis finds support in the presence of both an NDP kinase (NME6) and an NMP kinase (cytidine/uridine monophosphate kinase 2, CMPK2) within mitochondria (Xu *et al*, 2008). Alternatively, it is possible that CTP is continuously converted to CMP in its role as a cofactor in mitochondrial lipid metabolism (see below), and NME6 and CMPK2 are required to replenish CTP. The apparent absence of mitochondrial rN kinases suggests that rNs are not utilized by mitochondria.

In contrast to mitochondrial transcription, mtDNA replication was not affected by NME6 deficiency, which suggests that mitochondria can support DNA synthesis by importing pyrimidine DNA building blocks in the form of dNTPs via SLC25A33 and SLC25A36, despite the fact that dNTPs are present in the cytosol at concentrations several orders of magnitude below those of rNTPs (Fig 1—arrow C). It is important to note that changes in the cytosolic dNTP concentration have a direct impact on mitochondrial dNTP pools. This conclusion is supported by studies on SAMHD1, a deoxynucleoside triphosphohydrolase that directly degrades all four dNTPs to dNs. Inactivation of SAMHD1 leads to a shift from dNs toward dNTPs in the cytosol. As a consequence, the (mis)incorporation of rNTPs into mtDNA decreases (Wanrooij *et al*, 2020) and mtDNA copy number increases (Chong *et al*, 2022). Both of these changes indicate an elevation in mitochondrial dNTP pools can be caused by increased levels of cytosolic dNTPs, which is only expected if mitochondrial import can occur at the level of dNTPs.

An alternative import route for mitochondrial dNTP precursors involves the utilization of dNs (Fig 1—arrow D), which are phosphorylated to dNMPs by two dedicated mitochondrial dN kinases: TK2 and DGUOK. Both of these kinases are directly implicated in mtDNA depletion and deletion syndromes. NME6 is likely to play a crucial role in this pathway, as dNMPs require further phosphorylation to become dNTPs. While this route (Fig 1, arrow D) is not essential in actively dividing cells with high dNTP pools (TK2‐deficient mice exhibit normal embryonal development and early growth), it becomes essential in terminally differentiated cells (Akman *et al*, 2008; Zhou *et al*, 2008). Here, cytosolic dNTP pools are extremely low due to both reduced *de novo* production and the degradation of dNTPs to dNs by SAMHD1. Notably, TK2‐deficient mice rapidly develop weakness and succumb to death at the age of 2 to 4 weeks, highlighting the importance of nucleotide salvage in the maintenance of mitochondrial dNTP pools in postmitotic cells (Akman *et al*, 2008; Zhou *et al*, 2008).

One puzzling observation made by Grotehans *et al* is a significant but selective decrease in the concentration of mitochondrial dCTP, along with CTP, in NME6‐deficient cells. Why should only dCTP be reduced and not the other pyrimidine DNA building block, dTTP? One possibility is that when the CTP pool, which is the lowest among cellular rNTP pools, becomes too depleted in mitochondria, certain processes may utilize dCTP as its substitute. This could, for instance, occur in transcription or in metabolic reactions such as the synthesis of certain lipids. In the latter context, CTP is used in the conversion of phosphatidic acid to CDP‐diacylglycerol, which serves as an essential intermediate in the synthesis of phosphatidylglycerol, cardiolipin, and phosphatidylinositol (Blunsom *et al*, 2018). The notion that dCTP can participate in lipid metabolism is supported by the existence of dCDP‐ethanolamine and dCDP‐choline as well as the corresponding liponucleotides (Kennedy *et al*, 1959).

Another puzzling aspect is that the substantial decrease in dCTP does not seem to affect mtDNA copy number. It is possible that the dNTP concentrations that are present in mitochondria of actively dividing cells (with their significantly higher dNTP pools compared with nondividing cells) may not be limiting factors for mtDNA replication. It would be intriguing to investigate whether an equally large decrease in the dCTP pool would be observed in nondividing NME6 deficient cells, and if so, whether it would affect mtDNA replication.

Is the role of NME6 restricted to the maintenance of mitochondrial NTP pools? Mitochondrial transcript levels and the respiratory defect of NME6 knockout cells could be rescued by supplementing the cells with the pyrimidine nucleosides cytidine or uridine, both of which partly restore the depleted mitochondrial CTP levels found in these cells. However, nucleoside treatment was unable to fully correct the growth defect of the knockout cells, raising the interesting possibility of additional, nucleotide pool‐independent, functions of NME6. In line with this, the current study confirms previous reports of NME6 interacting with the guanine exchange factor RCC1L, which plays a role in mito‐ribosome assembly (Proust *et al*, 2021; preprint: Kramer *et al*, 2023). RCC1L is essential for maintaining normal levels of the mito‐ribosome and mitochondrial translation (Reyes *et al*, 2020). Although Grotehans *et al* did not find evidence of altered mito‐ribosomal protein levels, other studies have indicated dysregulation of mito‐ribosome assembly in NME6 knockout cells (Kramer *et al*, 2023). Further work is required to examine the impact of the NME6‐RCC1L interaction and to appreciate the full scope of NME6 functions in the mitochondria.

## Author contributions

**Paulina H Wanrooij:** Writing – original draft; writing – review and editing. **Andrei Chabes:** Writing – original draft; writing – review and editing.
